# Accuracy of Rapid Emergency Medicine Score and Sequential Organ Failure Assessment Score in predicting acute paraphenylenediamine poisoning adverse outcomes

**DOI:** 10.1007/s11356-022-24427-1

**Published:** 2022-12-03

**Authors:** Ghada N. El-Sarnagawy, Mona M. Ghonem, Marwa A. Abdelhameid, Omaima M. Ali, Asmaa M. Ismail, Doaa M. El Shehaby

**Affiliations:** 1 Department of Forensic Medicine and Clinical Toxicology, Faculty of Medicine, Tanta University, Al-Geish Street, Tanta City, Gharbia, 31527 Egypt; 2 Department of Internal Medicine, Faculty of Medicine, Aswan University, Aswan City, Egypt; 3 Department of Pediatrics, Faculty of Medicine, Aswan University, Aswan City, Egypt; 4 Department of Forensic Medicine and Clinical Toxicology, Faculty of Medicine, Assiut University, Assiut City, Egypt

**Keywords:** Paraphenylenediamine, Mortality, Complications, Rapid Emergency Medicine Score, Sequential Organ Failure Assessment, Acute respiratory failure, Acute renal failure, Rhabdomyolysis

## Abstract

Paraphenylenediamine (PPD) is a commonly used xenobiotic in hair dying, causing deleterious outcomes in acute poisoning. Although many epidemiological studies and case reports explained their clinical presentations and fatal consequences, no studies have evaluated the early determinants of adverse outcomes. Therefore, the present study aimed to assess the initial predictors of acute PPD poisoning adverse outcomes, focusing on the discriminatory accuracy of the Rapid Emergency Medicine Score (REMS) and Sequential Organ Failure Assessment (SOFA) score. A retrospective cohort study included all acute PPD-poisoned patients admitted to three Egyptian emergency hospitals from January 2020 to January 2022. Data was gathered on admission, including demographics, toxicological, clinical, scoring systems, and laboratory investigations. Patients were categorized according to their outcomes (mortality and complications). Ninety-seven patients with acute PPD poisoning were included, with a median age of 23 years, female predominance (60.8%), and suicidal intention (95.9%). Out of all patients, 25.77% died, and 43.29% had complicated outcomes. Respiratory failure was the primary cause of fatalities (10.30%), while acute renal failure (38.14%) was a chief cause of complications. The delay time till hospitalization, abnormal electrocardiogram, initial creatine phosphokinase, bicarbonate level, REMS, and SOFA scores were the significant determinants for adverse outcomes. The REMS exhibited the highest odds ratio (OR = 1.91 [95% confidence interval (CI): 1.41–2.60], *p* < 0.001) and had the best discriminatory power with the area under the curve (AUC) = 0.918 and overall accuracy of 91.8% in predicting mortality. However, the SOFA score had the highest odds ratio (OR = 4.97 [95% CI: 1.16–21.21], *p* = 0.001) and only yielded a significant prediction for complicated sequels with AUC = 0.913 and overall accuracy of 84.7%. The REMS is a simple clinical score that accurately predicts mortality, whereas the SOFA score is more practicable for anticipating complications in acute PPD-poisoned patients.

## Introduction

Paraphenylenediamine (PPD) is a para-nitroaniline derivative commercially manufactured by industrial companies (Akl and Alturki 2018). Upon contact with air, its color rapidly turns from white crystal to brown. As a result of the chemical properties of PPD as a color enhancer, it is extensively used in hair dyes to give hair a longer-lasting and naturally appearing black color (Abbas et al. 2017). It is known as kala-pathar in Pakistan and black stone in Arabic regions (Khaskheli et al. 2018). Additionally, PPD is used for other cosmetic purposes, such as temporary tattooing as well as printing inks, black rubber, oils, and photographic films (Panfili et al. 2017).

PPD acts as a pro-hapten that can be converted to multiple haptens through auto-oxidation reactions. These haptens are scavenged in the presence of adequate couplers such as resorcinol (Meyer and Fischer 2015). The maximum PPD content in standard hair dye formulas does not exceed 2% per 100 ml of color solution, making it less toxic for accidental poisoning in developed countries. In contrast, PPD concentrations in developing countries may reach 90% due to a lack of stringent regulations, resulting in serious consequences if intentionally ingested (Jain et al. 2011; Bhagavathula et al. 2019). Additionally, the toxicological potential of PPD commercial formulations is caused not only by high PPD concentrations but also by the illegal use of dye without enough couplers (Meyer and Fischer 2015).

Accordingly, PPD is considered a new suicidal trend following pesticides due to its low price, potent toxicity, and easy availability, especially in East Africa, the Indian subcontinent, and Middle East countries (Gude et al. 2012)**.** A systematic review and meta-analysis by Bhagavathula et al. (2019) recorded that PPD accounted for 93.5% of suicidal poisoning. Substantially, PPD is implicated in 35% of suicidal fatalities in Sudan, according to large-scale study (Elgamel and Ahmed 2013).

After oral ingestion, PPD is rapidly absorbed by gastrointestinal (GIT) mucous membranes into blood (Dawood et al. 2015). Afterward, PPD undergoes multiple reaction pathways, including activation and detoxification. Based on its hydrogen donor property, PPD is metabolized by the hepatic cytochrome P450 (CYP450) peroxidase enzyme through one-electron oxidation to a cation free radical to form a reactive benzoquinone diamine that can destroy muscle cells by membrane lipid peroxidation, causing skeletal and cardiac muscle necrosis (Khaskheli et al. 2018). Subsequently, the benzoquinone diamine is oxidized to Brandowaski’s base (BB), a trimer triggering anaphylaxis and mutation (Jain et al. 2011). Likewise, the CYP450 has also been linked to allergic contact dermatitis in cutaneous PPD exposure by excessive BB-inducing immune-stimulatory response in human keratinocytes (So et al. 2011). Another PPD activation step is through CYP450 oxidation to *N*-hydroxylamines, which can finally be transformed by O-acetylation or sulfation into reactive nitrenium ions, inducing DNA damage and triggering bladder cancer (Kumar et al. 2021). However, an in vitro study demonstrated a lack of evidence for PPD oxidative metabolism by CYP450 to form the potentially carcinogenic N-hydroxylamine compounds (Stanley et al. 2005). Furthermore, PPD releases free aminyl radicals through extrahepatic activation, resulting in further toxic oxidative stress effects (Eissa et al. 2021).

However, PPD undergoes another main detoxification pathway by *N*-acetyltransferase (NAT) enzymes, including (NAT-1) in the skin and (NAT-2) in the liver and gut to form *N*-acetyl-p-phenylenediamine (MAPPD) and *N*, *N*′-diacetyl-p-phenylenediamine (DAPPD) metabolites that are excreted in the urine (Hong et al. 2016). Therefore, in acute PPD exposure, a saturation of detoxification enzymes NAT1 and NAT2 results in the excretion of un-metabolized PPD and urochrome compounds, causing dark urine (Hooff et al. 2011).

Many studies have reported that the different enzyme genotypes or phenotypes involved in arylamine activation and/or detoxification may alter the carcinogenic risk of PPD (Gago-Dominguez et al. 2003). Although there is an increased incidence of PPD-inducing carcinogenicity in experimental studies, the likelihood of cancer after PPD exposure in humans is still controversial (Hong et al. 2016).

Patients can exhibit various symptoms depending on the route and amount of PPD exposure (Manzoor et al. 2017). A small oral dose of 3 g of PPD causes systemic manifestations, while a dose of 7 to 10 g can be lethal within the first 24 h (Senthilkumaran et al. 2019). Although skin irritation and contact dermatitis are the common topical effects, severe systemic toxicity usually ensues after ingesting large PPD doses (Khan et al. 2018). Clinically, patients are presented with anaphylactic signs reactions, including facial and mouth swelling, which may extend to the larynx, causing asphyxia and respiratory distress (Ali et al. 2020). Besides, other consequences like rhabdomyolysis, acute hepatitis, acute renal failure, myocarditis, seizures, shock, and even death may occur (Shalaby et al. 2010). Although angioneurotic edema is the leading cause of death within the first 24 h, acute renal failure is the dominant sequelae developed within the first week (Sakuntala et al. 2014; Haider et al. 2018).

Currently, no specific antidote is available for acute PPD poisoning; hence, treatment is mainly supportive with collaboration with other specialists for urgent tracheostomy in upper airway obstruction and hemodialysis for acute renal failure cases (Khan et al. 2016; Tanweer et al. 2018).

Scoring systems have been constructed to objectively assess critically ill patients’ severity and outcomes (Pellathy et al. 2021). Various scores depending on primary clinical data are used in emergency settings, such as Rapid Acute Physiology Score (RAPS), Simple Clinical Score (SCS), Rapid Emergency Medicine Score (REMS), and Modified Early Warning Score (MEWS) (Brabrand et al. 2010; El-Sarnagawy et al. 2022a). Alternatively, other reliable scores like Sequential Organ Failure Assessment (SOFA) and Multiple Organ Dysfunction Score (MODS) are preferred for better assessment of organ function complications in critical illness (Bota et al. 2002).

Despite the widespread use of scoring systems for anticipating various poisoning outcomes, limited published studies have evaluated these systems in predicting acute PPD poisoning consequences. Therefore, the study’s goal was to outline the predictors of mortality and adverse sequels in patients with acute PPD poisoning at three Egyptian emergency hospitals, highlighting the predictive accuracy of the REMS and SOFA scores. Subsequently, early prediction of life-threatening and delayed PPD sequels provides quick early intervention and follow-up for high-risk patients as well as ensures the optimal use of hospital resources.

## Patients and methods

### Study design and setting

A multicenter retrospective observational study was conducted on the medical records of all adult patients with acute PPD poisoning admitted to emergency hospitals of three Egyptian Faculties of Medicine (Tanta, Assiut, and Aswan) over two past years from January 2020 to January 2022.

### Inclusion criteria

All adult patients aged 18 years or older of both sexes with acute PPD poisoning were enrolled. Acute PPD poisoning can be defined as exposure to a massive PPD dose within a short period (less than 24 h) in a single instance (Dawood et al. 2015; Tefera and Teferi 2020). Our diagnosis depends on a history of acute PPD exposure, supported by characteristic manifestations and the presence of a hair dye container.

### Exclusion criteria

We excluded twenty-three patients with chronic comorbidities or organ dysfunctions (e.g., cardiovascular, respiratory, renal, and hepatic diseases, as well as infection and cancer) from reviewing the medical records of patients’ files. Additionally, patients with unclear diagnoses, mixed congestion, and missing data were excluded from this study.

### Sample size

**Fig. 1:**
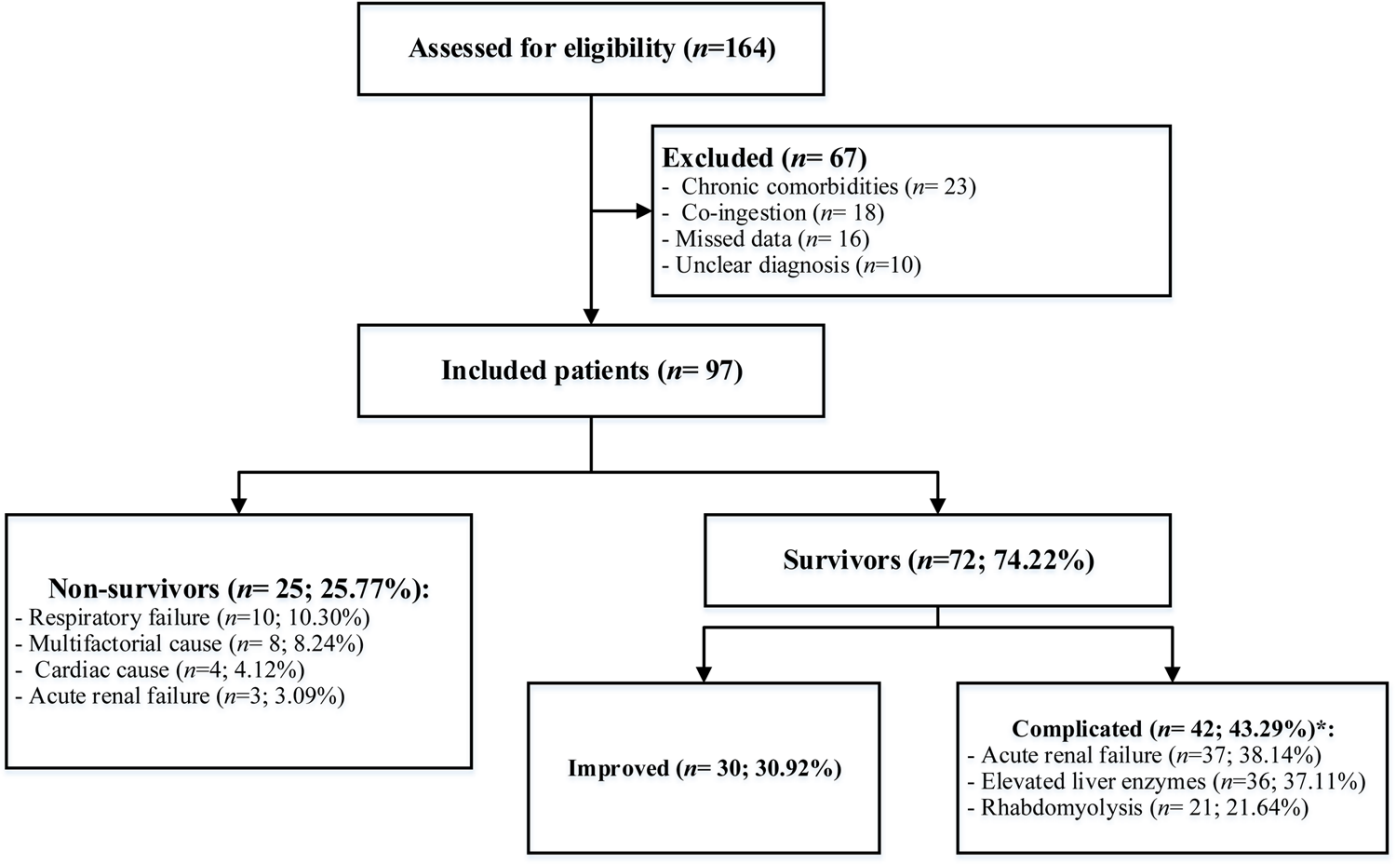
Flow chart of the studied acute paraphenylenediamine-poisoned patients, including the causes of death and types of complications; *: Some patients had more than one complication

To include the highest number of patients, we enrolled all acutely PPD-poisoned patients according to the eligibility criteria. Out of patients diagnosed with acute PPD ingestions (*n* = 164) in the study period, only 97 patients were eligible and included after eliminating 67 patients, as presented in Fig. 1.

### Ethical considerations

This study was performed according to the Declaration of Helsinki and after the agreement of the Research Ethics Committee of Faculties of Medicine (Tanta, Assiut, and Aswan) Universities with approval numbers: 35015/11/21, 17300942, and Asw.u.689/11/22, respectively. A code number for each patient was given to maintain patient confidentiality, making statistical data analysis anonymous. Due to the retrospective study design, the written informed consent was waived.

### Data collection

#### Demographic and clinical data

From the patients’ medical records, we gathered the demographic profiles of the patients (age and gender) and poisoning-related data (the mode of poisoning, route of exposure, the delay time before hospital admission, and the hospitalization period). The initial presenting symptoms were documented, including gastrointestinal, respiratory, cardiac, and renal manifestations. Additionally, the baseline vital data, including pulse, systolic blood pressure [SBP], diastolic blood pressure [DBP], respiratory rate, and temperature, were recorded.

#### Scoring systems

On hospital admission, the included acutely PPD-poisoned patients were evaluated using the Poisoning Severity Score (PSS) into four grades of severity (grade 0: asymptomatic, grade 1: minor symptoms, grade 2: moderate symptoms, and grade 3: severe symptoms) (Persson et al. 1998).

Similarly, the two studied scores were assessed at the initial presentation time. The Rapid Emergency Medicine Score (REMS) was calculated by adding age value to the other five variables, including pulse rate, mean arterial pressure, respiratory rate, Glasgow Coma Scale (GCS), and peripheral oxygen saturation. Each variable ranges from 0 to 4 except for age, scoring 0 to 6. The total REMS ranged from 0 to 26, and the higher value indicates a bad prognosis (Olsson et al. 2004).

Furthermore, we assessed the Sequential Organ Failure Assessment (SOFA) score, which contains parameters of respiratory function (PO_2_ / FIO_2_), hepatic function (serum bilirubin), renal function (serum creatinine), cardiovascular function (mean arterial pressure and the administrated vasopressors dose), hematologic function (platelet count), and neurologic function (GCS). Each organ was scored from 0 to 4 points, yielding a total score value ranging from 0 (normal value) to 24 (the worst value) (Vincent et al. 1998).

#### Investigations and electrocardiography

Initial laboratory investigation results were recorded from patients’ files. Arterial blood gases (pH, PaO_2_, PCo_2_, and serum bicarbonate [HCO_3_]) were analyzed by pHOx plus L using Stat profile calibrator cartridge C from Nova Biomedical GmbH, Germany (catalog number: 34086). Aspartate transaminase [AST], alanine transaminase [ALT], blood urea, and serum creatinine were measured by the Konelab Prime 60i apparatus using kits obtained from Thermo Fisher Scientific-Finland (catalog numbers: 981769, 981771, 981820, and 981811, respectively). Besides, serum creatine phosphokinase (CPK) was assayed by a kinetic method using CK-NAC-LQ kits from Spainreact, Spain (catalog number: 41250). Additionally, an electrocardiogram (ECG) was captured to assess any conduction abnormalities.

#### Treatment and in-hospital outcomes

Due to the lack of a precise antidote, the management of acute PPD is mainly supportive. In cases of ingestion within 2 h, gastric lavage was performed, followed by activated charcoal administration of 1 g/kg. In hypoxia cases (SaO_2_ < 90%), oxygen was administered. Intravenous corticosteroids, calcium gluconate, and antihistaminic were administered for all cases with angioneurotic edema. Alternatively, emergency tracheostomy was applied in cases presented with severe laryngeal edema, stridor, and falling oxygen saturation. All patients received adequate fluid therapy of 2–3 L/day to maintain hydration. In persistent hypotensive patients, vasopressors and inotropic (noradrenaline/ dopamine) were administered, depending on clinical status. Forced alkaline diuresis by sodium bicarbonate was done to avoid acute renal injury secondary to rhabdomyolysis. However, hemodialysis was performed for cases with acute renal shutdown, resistant hyperkalemia, and unimproved metabolic acidosis.

The included patients were grouped according to the in-hospital mortality into survivors and non-survivors. After then, the survivors were subgrouped according to adverse sequels into complicated and improved. The possible causes of death were recorded, including respiratory failure, acute renal failure, cardiac arrhythmia, and multiorgan failure. The recorded complicated consequences included acute kidney injury (elevated serum creatinine > 0.3 mg/dL in 2 days or 1.5 times from baseline weekly), elevated liver enzymes (elevated transaminases up to 3 times the upper reference value), and rhabdomyolysis (elevated CPK > 1500U/L) (Malakouti et al. 2017; Ali et al. 2020; Gayathri et al. 2021).

### Statistical analysis

The used analytical program was Statistical Package for Social Sciences (IBM SPSS Statistics) for Windows, version 26 (IBM Corp., Armonk, NY, USA). Qualitative variables were presented by counts and percentages. The association of the studied outcomes with categorical variables was tested using Pearson’s chi-square test for independence test, Fisher-Freeman-Halton exact test, and Fisher’s exact test. As for the quantitative variables, the Shapiro–Wilk test was performed to determine their distribution. Variables with normal distributions were represented as mean ± standard deviation (SD) and were tested using the independent samples *t*-test. Data that did not correspond to the normal distribution were displayed as the median and interquartile range (expressed as the 25th–75th percentiles) and analyzed using the Mann–Whitney test. Binomial logistic regression analysis was conducted to determine the contribution of clinically relevant predictors to mortality and complications. The performance of REMS and SOFA scores was assessed by analysis of the receiver-operating characteristics (ROC) curve. A *p*-value < 0.05 was adopted to interpret the significance of statistical tests.

## Results

### Patients enrollment, grouping, and characteristics

Ninety-seven patients with acute PPD poisoning met the eligibility criteria and were included in this study. The highest percentage of acute PPD were females (60.8%) with a median age of 23 (IQR: 19–31). The dominant presenting symptoms were gastrointestinal, cervicofacial edema, and dark urine (80.4%, 50.5%, and 49.5%, respectively). Ingestion is the only observed route, and most patients (95.9%) intentionally ingested PPD for suicidal attempts. The highest percentage of patients (46.4%) had moderate PSS with medians of one and two for REMS and SOFA scores, respectively (Table 1).

**Table 1:** Comparison of demographic and clinical data, studied scores, and hospital stay between survived and non-survived acute paraphenylenediamine-poisoned patients (*n* = 97)

Characteristics		Non-survivors *n* = 25	Survivors *n* = 72	*p-* value	Total *n* = 97
Age (years)	Median [IQR] (Min–max)	28.0 [20.0–43.0] (18.0–65.0)	22.0 [18.5–29.0] (18.0–75.0)	0.042*^a^	23.0 [19.0–31.0] (18.0–75.0)
Sex	Male	15 (60.0%)	23 (31.9%)	0.013*^b^	38 (39.2%)
	Females	10 (40.0%)	49 (68.1%)		59 (60.8%)
Mode	Accidental	0 (0.0%)	4 (5.6%)	0.570 ^c^	4 (4.1%)
	Suicidal	25 (100.0%)	68 (94.4%)		93 (95.9%)
Delay	≤ 6hs	0 (0.0%)	44 (61.1%)	< 0.001*^c^	44 (45.4%)
	> 6hs	25 (100.0%)	28 (38.9%)		53 (54.6%)
Cervicofacial edema		22 (88.0%)	27 (37.5%)	< 0.001*^c^	49 (50.5%)
Laryngeal edema		16 (64.0%)	20 (27.8%)	0.001*^b^	36 (37.1%)
Dyspnea		19 (76.0%)	15 (20.8%)	< 0.001*^b^	34 (35.1%)
Cyanosis		10 (40.0%)	1 (1.4%)	< 0.001*^b^	11 (11.3%)
Stridor		16 (64.0%)	21 (29.2%)	0.002*^b^	37 (38.1%)
Gastrointestinal manifestations		16 (64.0%)	62 (86.1%)	0.016*^b^	78 (80.4%)
Neuropathy and myalgia		5 (20.0%)	7 (9.7%)	0.179 ^c^	12 (12.4%)
Shock		12 (48.0%)	2 (2.8%)	< 0.001*^c^	14 (14.4%)
Dark urine		13 (52.0%)	35 (48.6%)	0.770 ^b^	48 (49.5%)
Oliguria		9 (36.0%)	32 (44.4%)	0.461^b^	41 (42.3%)
Pulse (beat/min)	Mean ± SD (Min–max)	102.0 ± 22.2 (67.0–150.0)	88.4 ± 13.0 (66.0–120.0)	0.007* ^d^	91.9 ± 16.8 (66.0–150.0)
SBP (mmHg)	Mean ± SD (Min–max)	100.3 ± 27.8 (60.0–170.0)	122.7 ± 21.2 (80.0–170.0)	< 0.001* ^d^	116.9 ± 24.9 (60.0–170.0)
DBP (mmHg)	Mean ± SD (Min–max)	63.5 ± 15.8 (40.0–100.0)	75.4 ± 11.8 (56.0–100.0)	< 0.001* ^d^	72.4 ± 13.9 (40.0–100.0)
RR (cycle/min)	Mean ± SD (Min–max)	30.4 ± 8.8 (18.0–48.0)	21.5 ± 4.3 (16.0–38.0)	< 0.001* ^d^	23.8 ± 6.9 (16.0–48.0)
Temperature (°C)	Mean ± SD (Min–max)	36.9 ± 0.7 (36–38.2)	37.1 ± 0.5 (36.2–38.5)	0.126 ^d^	37.1 ± 0.5 (36.0–38.5)
REMS	Median [IQR] (Min–max)	7 [4–10] (0–16)	1 [0–2] (0–4)	< 0.001*^a^	1 [0–3] (0–16)
SOFA score	Median [IQR] (Min–max)	5 [4–6] (2–11)	1 [1–4] (0–7)	< 0.001*^a^	2 [1–5] (0–11)
PSS grades	Asymptomatic	0 (0.0%)	5 (6.9%)	< 0.001*^c^	5 (5.2%)
	Minor	0 (0.0%)	25 (34.7%)		25 (25.8%)
	Moderate	11 (44.0%)	34 (47.2%)		45 (46.4%)
	Severe	14 (56.0%)	8 (11.1%)		22 (22.7%)
Hospital stay (days)	Median [IQR] (Min–max)	2 [2–3] (1–6)	6 [4–7] (2–14)	< 0.001*^a^	4 [3–7] (1–14)

*n* number, *IQR* interquartile range, *Min* minimum, *Max* maximum, *SD* standard deviation, *SBP* systolic blood pressure, *DBP* diastolic blood pressure, *RR* respiratory rate, *REMS* Rapid Emergency Medicine Score, *SOFA* Sequential Organ Failure Assessment, *PSS* Poisoning Severity Score

^a^Mann-Whitney test

^b^Pearson’s chi-square test

^c^Fisher’s or Fisher-Freeman-Halton exact tests

^d^Independent samples *t*-test

^*^Significant at *p* < 0.05

Out of enrolled patients, 25.77% (25 patients) died, and 74.22% (72 patients) survived. Among the survivors, 42 (43.29%) patients had complicated outcomes, and thirty (30.92%) patients completely recovered. Respiratory failure was a primary cause of fatality (*n* = 10; 10.30%). In contrast, acute renal failure was the chief cause of complications (*n* = 37; 38.14%), as shown in Fig. 1.

### Comparison between survivors’ and non-survivors’ characteristic data

Table 1 shows that the non-survivors had a significantly higher median age than survivors (Median: 28 vs. 22, *p* = 0.042), with male predominance (60%). All non-survivors were admitted after 6 h post-ingestion. Most non-survivors presented significantly with cervicofacial edema (88%), dyspnea (76%), laryngeal edema (64%), stridor (64%), shock (48%), and cyanosis (40%) than survivors (*p* < 0.05). Compared to survivors, the non-survivors had significantly lower means of SBP (100.3 ± 27.8 vs. 122.7 ± 21.2 mmHg, *p* < 0.001) and DBP (63.5 ± 15.8 vs. 75.4 ± 11.8 mmHg, *p* < 0.001) and significantly higher means of pulse (102 ± 22.2 vs. 88.4 ± 13.0 beat/min*, p* = 0.007) and respiratory rates (30.4 ± 8.8 vs. 21.5 ± 4.3 cycle/min, *p* < 0.001). The median values of REMS and SOFA scores were significantly higher in non-survivors than survivors (*p* < 0.001 for each). Furthermore, more than half the non-survivors (56%) had significantly severe PSS, whereas 47.2% of survivors had moderate PSS (*p* < 0.001).

The non-survived patients had a significantly lower PaO_2_ than the survivors (62.2 ± 12.5 vs. 84.9 ± 9.3 mmHg, *p* < 0.001). Additionally, the median value of CPK was elevated significantly among non-survivors than survivors (1500 vs. 335 U/L, *p* = 0.009). Abnormal ECG findings were recorded considerably in 40% of non-survivors; meanwhile, the majority of survivors (94.4%) had normal ECG (*p* < 0.001). However, there were no significant differences between survivors and non-survivors regarding pH, PaCO_2_, HCO_3_, AST, ALT, blood urea, and serum creatinine (*p* > 0.05), as illustrated in Table 2.

**Table 2:** Comparison of laboratory investigations and electrocardiographic findings between survivors and non-survivors of acute paraphenylenediamine-poisoned patients (*n* = 97)

Characteristics		Non- survivors *n* = 25	Survivors *n* = 72	*p-*value	Total *n* = 97
pH	Mean ± SD (Min–max)	7.33 ± 0.04 (7.21–7.45)	7.33 ± 0.09 (7.10–7.50)	0.901^a^	7.33 ± .08 (7.10–7.50)
PaO_2_ (mmHg)	Mean ± SD (Min–max)	62.2 ± 12.5 (40.0–88.0)	84.9 ± 9.3 (55.0–98.0)	< 0.001*^a^	79.1 ± 14.2 (40.0–98.0)
PaCO_2_ (mmHg)	Mean ± SD (Min–max)	29.1 ± 10.4 (18.0–60.1)	31.6 ± 7.6 (16.0–50.8)	0.203 ^a^	30.9 ± 8.4 (16.0–60.1)
HCO_3_ (mEq/L)	Mean ± SD (Min–max)	18.2 ± 5.0 (9.0–32.9)	19.7 ± 5.5 (8.0–28.1)	0.929 ^a^	19.3 ± 5.4 (8.0–32.9)
AST (U/L)	Median [IQR] (Min–max)	100.0 [35.0–600.0] (18.0–3800.0)	91.5 [35.0–289.5] (14.0–1985.0)	0.629 ^b^	95.0 [35.0–295.0] (14.0–3800.0)
ALT (U/L)	Median [IQR] (Min–max)	50.0 [39.0–290.0] (8.0–6300.0)	149.5 [37.5–451.5] (12.0–2200.0)	0.523 ^b^	111.0 [39.0–450.0] (8.0–6300.0)
Blood urea (mg/dL)	Median [IQR] (Min–max)	37.0 [25.0–65.0] (18.0–180.0)	46.0 [26.5–158.0] (15.0–280.0)	0.220 ^b^	43.0 [26.0–112.0] (15.0–280.0)
Serum Creatinine (mg/dL)	Median [IQR] (Min–max)	1.40 [1.00–2.30] (0.60–5.10)	1.45 [.80–4.05] (0.34–8.00)	0.859 ^b^	1.40 [0.80–2.90] (0.34–8.00)
Serum CPK (U/L)	Median [IQR] (Min–max)	1500.0 [310.0–7000.0] (50.0–11,570.0)	335.0 [150.0–1650.0] (70.0–6700.0)	0.009*^b^	500.0 [150.0–2300.0] (50.0–1157.0)
ECG	Normal	15 (60.0%)	68 (94.4%)	< 0.001* ^c^	83 (85.6%)
	Abnormal	10 (40.0%)	4 (5.6%)		14 (14.4%)

*n* number, *SD* standard deviation, *Min* minimum, *Max* maximum, *IQR* interquartile range, *PaO*_*2*_ partial arterial oxygen pressure, *PaCO*_*2*_ partial arterial carbon dioxide pressure, *HCO*_*3*_ bicarbonate, *AST* aspartate aminotransferase, *ALT* alanine transaminase, *CPK* creatine phosphokinase, *ECG* electrocardiography

^a^Independent samples *t*-test

^b^Mann-Whitney test

^c^Fisher’s exact test

^*^Significant at *p* < 0.05

### Comparison between complicated and improved patients’ characteristic data

Complications in acute PPD-poisoned patients were significantly associated with delayed admission after 6 h (*p* < 0.001), with no significant difference in age and sex. The most frequently reported symptoms in complicated patients were GIT manifestations (100%), dark urine (83.3%), and oliguria (76.2%). The mean SBP was significantly elevated in the complicated group than in the non-complicated patients (128.8 ± 19.5 vs. 114.3 ± 20.8 mmHg, *p* = 0.004). Although the median of the REMS score did not exhibit a significant difference (*p* = 0. 853), the SOFA score was significantly higher in complicated patients than in non-complicated (median values: 3 versus zero, *p* < 0.001). Furthermore, most complicated patients had moderate PSS (69%), whereas 66.7% of non-complicated patients had mild PSS (*p* < 0.001), as shown in Table 3.

**Table 3:** Comparison of demographics and clinical data, studied scores, and hospital stay among complicated and improved patients with acute paraphenylenediamine poisoning (*n* = 72)

Characteristics		Complicated *n* = 42	Improved *n* = 30	*p-*value	Total *n* = 72
Age (years)	Median [IQR] (Min–max)	23.0 [19.0–31.0] (18.0–42.0)	21.5 [18.0–26.0] (18.0–75.0)	0.203^a^	23.0 [19.0–31.0] (18.0–75.0)
Sex	Male	11 (26.2%)	12 (40.0%)	0.215 ^b^	38 (39.2%)
	Females	31 (73.8%)	18 (60.0%)		59 (60.8%)
Mode	Accidental	0 (0.0%)	4 (7.3%)	0.131^c^	4 (4.1%)
	Suicidal	42 (100.0%)	51 (92.7%)		93 (95.9%)
Delay	≤ 6 h	15 (35.7%)	29 (96.7%)	< 0.001*^c^	44 (45.4%)
	> 6 h	27 (64.3%)	1 (3.3%)		53 (54.6%)
Cervicofacial edema		15 (35.7%)	12 (40.0%)	0.711^b^	49 (50.5%)
Laryngeal edema		12 (28.6%)	8 (26.7%)	0.859 ^b^	36 (37.1%)
Dyspnea		10 (23.8%)	5 (16.7%)	0.462 ^b^	34 (35.1%)
Cyanosis		1 (2.4%)	0 (0.0%)	1.000 c	11 (11.3%)
Stridor		14 (33.3%)	7 (23.3%)	0.357 ^b^	37 (38.1%)
Gastrointestinal manifestations		42 (100.0%)	20 (66.7%)	< 0.001*^b^	78 (80.4%)
Neuropathy and myalgia		6 (14.3%)	1 (3.3%)	0.227^c^	12 (12.4%)
Shock		1 (2.4%)	1 (3.3%)	1.000 ^c^	14 (14.4%)
Dark urine		35 (83.3%)	0 (0.0%)	< 0.001*^c^	48 (49.5%)
Oliguria		32 (76.2%)	0 (0.0%)	< 0.001*^c^	41 (42.3%)
Pulse (beat/min)	Mean ± SD (Min–max)	87.9 ± 13.3 (66.0–120.0)	89.0 ± 12.6 (68.0–120.0)	0.740 ^d^	88.4 ± 13.0 (66.0–120.0)
SBP (mmHg)	Mean ± SD (Min–max)	128.8 ± 19.5 (90.0–170.0)	114.3 ± 20.8 (80.0–150.0)	0.004* ^d^	122.7 ± 21.2 (80.0–170.0)
DBP (mmHg)	Mean ± SD (Min–max)	77.1 ± 12.1 (56.0–100.0)	73.1 ± 11.2 (60.0–98.0)	0.159 ^d^	75.4 ± 11.8 (56.0–100.0)
RR (cycle/min)	Mean ± SD (Min–max)	20.8 ± 3.8 (16.0–30.0)	22.5 ± 4.8 (16.0–38.0)	0.093 ^d^	21.5 ± 4.3 (16.0–38.0)
Temperature (°C)	Mean ± SD (Min–max)	37.1 ± 0.5 (36.3–37.9)	37.1 ± 0.5 (36.2–38.5)	0.876 ^d^	37.1 ± 0.5 (36.2–38.5)
REMS	Median [IQR] (Min–max)	1 [0–2] (0–4)	1 [0–1] (0–4)	0.853 ^a^	1 [0–2] (0–4)
SOFA score	Median [IQR] (Min–max)	3 [2–5] (0–7)	0 [0–1] (0–2)	< 0.001*^a^	1 [1–4] (0–7)
PSS grades	Asymptomatic	0 (0.0%)	5 (16.7%)	< 0.001*^c^	5 (6.9%)
	Minor	5 (11.9%)	20 (66.7%)		25 (34.7%)
	Moderate	29 (69.0%)	5 (16.7%)		34 (47.2%)
	Severe	8 (19.0%)	0 (0.0%)		8 (11.1%)
Hospital stay (days)	Median [IQR] (Min–max)	7 [6–8] (3–14)	4 [3–4] (2–7)	< 0.001*^a^	6 [4–7] (2–14)

*n* number, *IQR* interquartile range, *Min* minimum, *Max* maximum, *SD* standard deviation, *SBP* systolic blood pressure, *DBP* diastolic blood pressure, *RR* respiratory rate, *REMS* Rapid Emergency Medicine Score, *SOFA* Sequential Organ Failure Assessment, *PSS* Poisoning Severity Score

^a^Mann-Whitney test

^b^Pearson’s chi-square test

^c^Fisher’s or Fisher-Freeman-Halton exact tests

^d^Independent samples *t*-test

^*^Significant at *p* < 0.05

Table 4 reveals that the complicated patients had significantly lower mean values of pH, PaO_2_, PaCO_2_, and HCO_3_ than those of non-complicated patients (*p* < 0.001, 0.023, < 0.001, and < 0.001, respectively). However, serum AST, ALT, blood urea, serum creatinine, and serum CPK level were significantly higher in complicated patients than in non-complicated patients (*p* < 0.001 for each).

**Table 4:** Comparison of laboratory investigations and electrocardiographic findings among complicated and improved acute paraphenylenediamine-poisoned patients (*n* = 72)

Characteristics		Complicated *n* = 42	Improved *n* = 30	*p*-value	Total *n* = 72
pH	Mean ± SD (Min–max)	7.28 ± 0.09 (7.10–7.43)	7.39 ± 0.05 (7.25–7.50)	< 0.001*^a^	7.33 ± .09 (7.10–7.50)
PaO_2_ (mmHg)	Mean ± SD (Min–max)	83.0 ± 10.9 (55.0–95.0)	87.6 ± 5.6 (78.0 98.0)	0.023*^a^	84.9 ± 9.3 (55.0–98.0)
PaCO_2_ (mmHg)	Mean ± SD (Min–max)	29.0 ± 8.6 (16.0–50.8)	35.2 ± 3.4 (26.0–47.0)	< 0.001*^a^	31.6 ± 7.6 (16.0–50.8)
HCO_3_ (mEq/L)	Mean ± SD (Min–max)	17.0 ± 5.5 (8.0–28.1)	23.3 ± 2.8 (16.0–28.0)	< 0.001*^a^	19.7 ± 5.5 (8.0–28.1)
Serum Na (mmol/L)	Mean ± SD (Min–max)	137.8 ± 3.4 (132.0–145.0)	139.0 ± 4.5 (133.0–153.0)	0.188 ^a^	138.3 ± 3.9 (132.0–153.0)
Serum K (mmol/L)	Mean ± SD (Min–max)	4.52 ± 0.89 (3.40–6.40)	3.88 ± 0.41 (3.10–4.78)	< 0.001*^a^	4.26 ± 0.79 (3.10–6.40)
AST (U/L)	Median [IQR] (Min–max)	277.0 [160.0–435.0] (15.0–1985.0)	35.0 [22.0–51.0] (14.0–115.0)	< 0.001*^b^	91.5 [35.0–289.5] (14.0–1985.0)
ALT (U/L)	Median [IQR] (Min–max)	404.5 [220.0–651.0] (19.0–2200.0)	32.5 [18.0–48.0] (12.0–128.0)	< 0.001*^b^	149.5 [37.5–451.5] (12.0–2200.0)
Blood urea (mg/dL)	Median [IQR] (Min–max)	121.5 [80.0–195.0] (16.0–280.0)	25.5 [18.5–33.0] (15.0–87.0)	< 0.001*^b^	46.0 [26.5–158.0] (15.0–280.0)
Serum Creatinine (mg/dL)	Median [IQR] (Min–max)	3.05 [1.90–4.60] (0.50–8.00)	0.77 [0.60–0.90] (0.34–1.30)	< 0.001*^b^	1.45 [0.80–4.05] (0.34–8.00)
Serum CPK (U/L)	Median [IQR] (Min–max)	1300.0 [400.0–4300.0] (80.0–6700.0)	145.0 [90.0–200.0] (70.0–1000.0)	< 0.001*^b^	335.0 [150.0–1650.0] (70.0–6700.0)
ECG	Normal	41 (97.6%)	27 (90.0%)	0.301^c^	68 (94.4%)
	Abnormal	1 (2.4%)	3 (10.0%)		4 (5.6%)

*n* number, *SD* standard deviation, *Min* minimum, *Max* maximum, *IQR* interquartile range, *PaO*_*2*_ partial arterial oxygen pressure, *PaCO*_*2*_ partial arterial carbon dioxide pressure, *HCO*_*3*_ bicarbonate, *AST* aspartate aminotransferase, *ALT* alanine transaminase, *CPK* creatine phosphokinase, *ECG* electrocardiography

^a^Independent samples *t*-test

^b^Mann-Whitney test

^c^Fisher’s exact test

^*^Significant at *p* < 0.05

### Treatment measures among patient groups

**Fig. 2:**
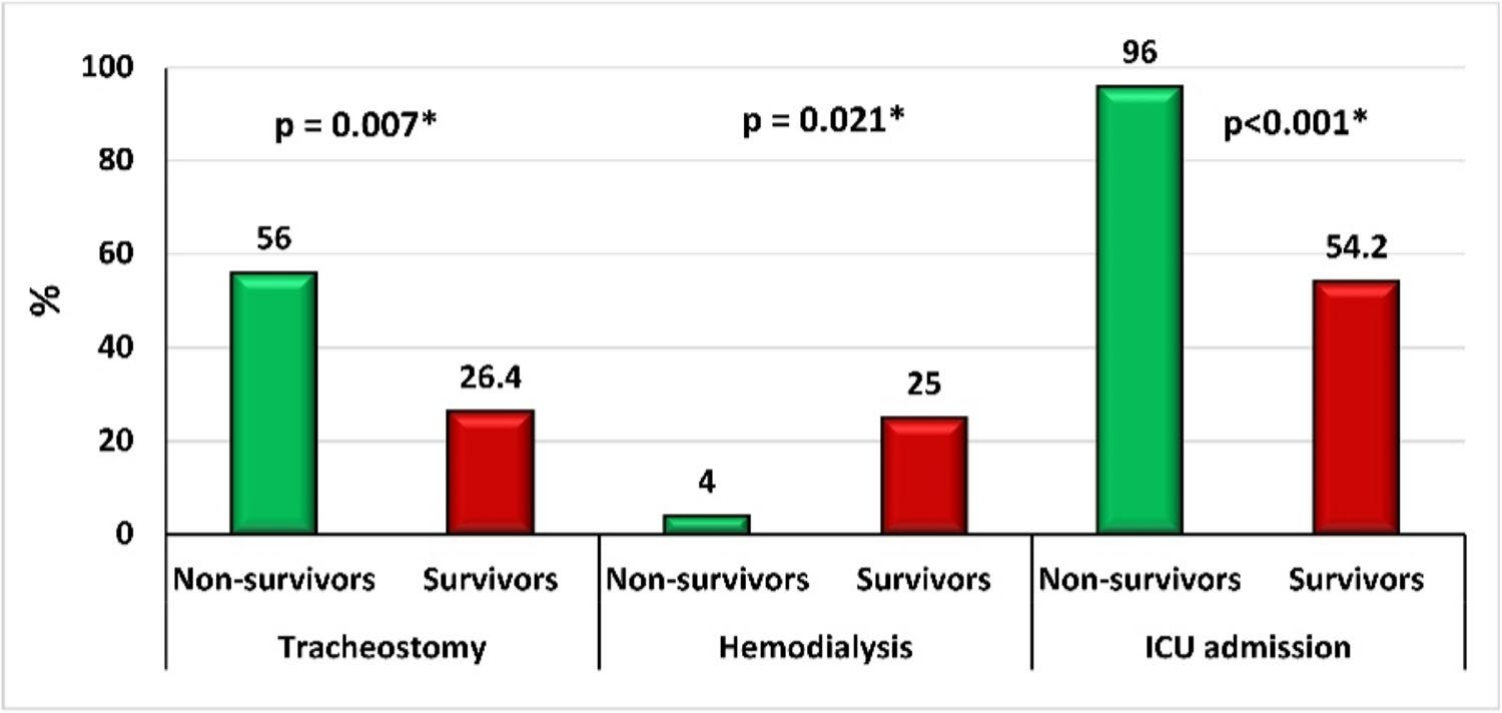
Comparison of tracheostomy, hemodialysis, and intensive care unit (ICU) admission between survivors and non-survivors in acute paraphenylenediamine-poisoned patients

Out of included patients, 34.0% underwent tracheostomy, 38.14% received hemodialysis, and 64.9% were admitted to the ICU. Ultimately, the majority of non-survivors were significantly admitted to ICU (96%) and underwent a tracheostomy (56%) than survivors (*p* < 0.001 and *p* = 0.007, respectively). However, hemodialysis was observed in a significantly higher percentage of survivors compared to non-survivors (25% vs. 4%, *p* = 0.021), as demonstrated in Fig. 2.

**Fig. 3:**
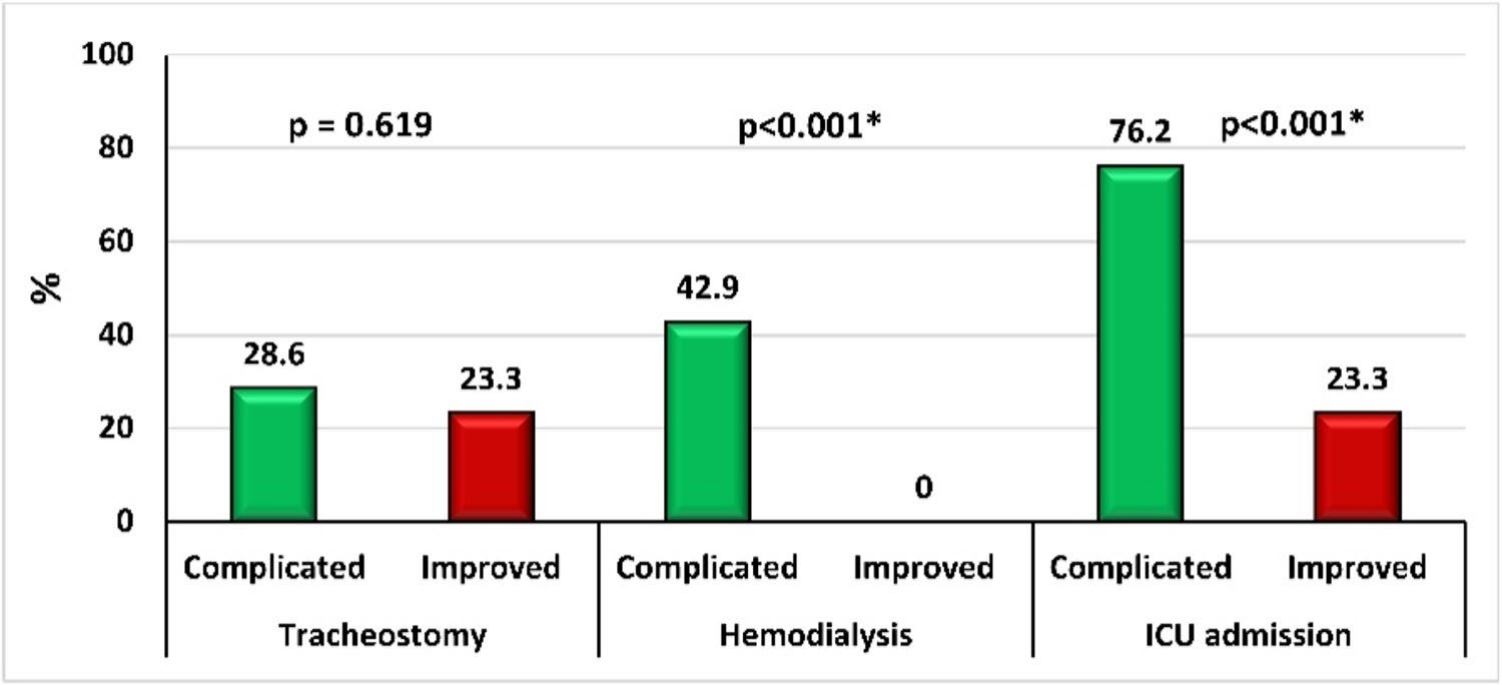
Comparison of tracheostomy, hemodialysis, and intensive care unit (ICU) admission between complicated and improved acute paraphenylenediamine-poisoned patients

Similarly, Fig. 3 depicts that—compared with improved patients—most patients with adverse sequels were significantly admitted to ICU (76.2% vs. 23.3%, *p* < 0.001) and received hemodialysis (42.9% vs. 0%, *p* < 0.001). Conversely, no significant difference was observed regarding tracheostomy between patients with and without complications (*p* = 0.619).

### Performance of the studied scores in predicting mortality and adverse sequels

Table 5 reveals the findings of univariate and multivariate regression analyses evaluating the significant variables affecting PPD adverse outcomes. Although delay time for admission, abnormal ECG, initial CPK level, as well as REMS and SOFA scores, were the significant determinants by univariate analysis, the REMS had the highest odds ratio for predicting mortality by multivariate analysis (OR = 1.91 [95% CI: 1.41–2.60], *p* < 0.001). Likewise, the delay time for admission, initial HCO_3_, CPK levels, and SOFA score could significantly predict complications by univariate analysis. However, based on multivariate analysis, the SOFA score was the most significant predictor of complications (OR = 4.97 [95% CI: 1.16–21.21], *p* = 0.001).

**Table 5:** Univariate and multivariate binary logistic regression analyses for significant variables affecting mortality and complications in patients with acute paraphenylenediamine poisoning

Outcome	Predictors	Univariate analysis		Multivariate model	
		*p*	OR (95% CI)	*p*	OR(95% CI)
Mortality	Delay (hours)	< 0.001*	1.78 (1.38–2.29)	< 0.001*	0.65 (0.53–0.80)
	Abnormal ECG	< 0.001*	11.33 (3.12–41.06)	0.961	0.95 (0.14–6.14)
	Serum CPK	0.002*	1.000 (1.00–1.00)	0.221	1.00 (1.00–1.00)
	REMS	< 0.001*	2.76 (1.69–4.49)	< 0.001*	1.91 (1.41–2.60)
	SOFA score	< 0.001*	1.94 (1.45–2.60)	0.372	1.20 (0.80–1.80)
Complications	Delay (hours)	< 0.001*	2.36 (1.58–3.53)	0.332	1.33 (0.74–2.36)
	HCO_3_	< 0.001*	0.72 (0.61–0.85)	0.030*	0.80 (0.71–0.91)
	Serum CPK	0.003*	1.00 (1.00–1.00)	0.093	1.00 (0.99–1.00)
	SOFA score	< 0.001*	6.91(2.59–18.40)	0.001*	4.97 (1.16–21.21)

*CI* confidence interval, *OR* odds ratio, *REMS* Rapid Emergency Medicine Score, *SOFA* Sequential Organ Failure Assessment, *ECG* electrocardiography, *CPK* creatine phosphokinase, *HCO*_*3*_ bicarbonate; *significant at *p* < 0.05

**Fig. 4:**
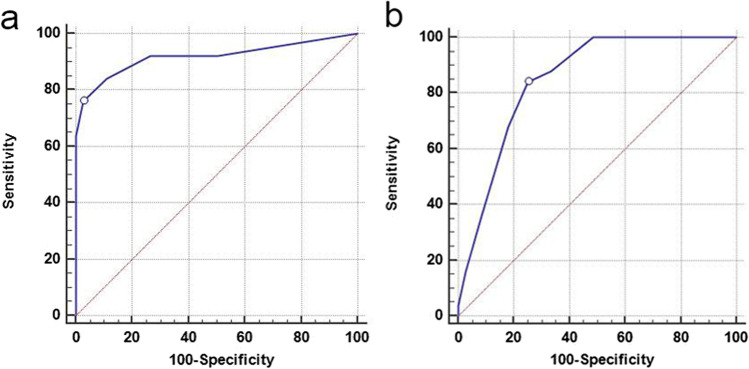
Receiver-operating characteristics curves of REMS (**a**) and SOFA score (**b**) for predicting mortality of acute paraphenylenediamine-poisoned patients. REMS and SOFA scores exhibit comparable AUC (0.918 and 0.850, respectively). However, the REMS at a cutoff value > 3 conveys higher specificity (97.2%) and overall accuracy (91.8%) compared to the SOFA score

**Fig. 5:**
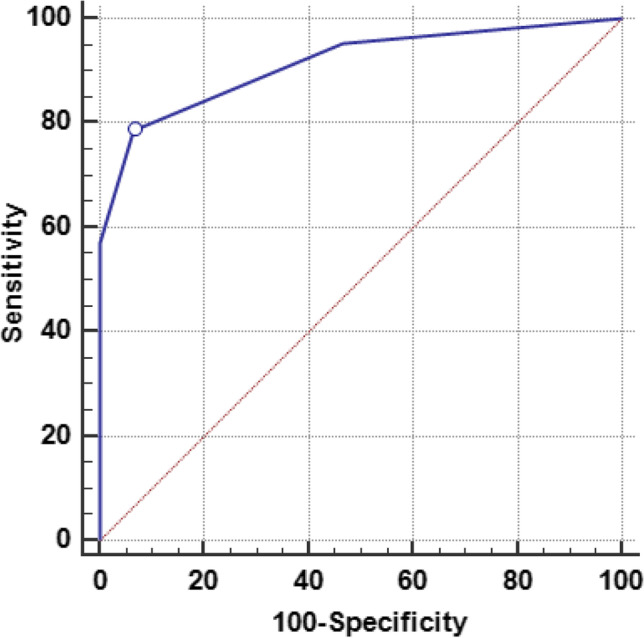
Receiver-operating characteristics curves of SOFA score for predicting complications of acute paraphenylenediamine-poisoned patients. The SOFA score at a cutoff value > 1 can significantly predict complications with excellent performance (AUC = 0.913), sensitivity (78.57%) and specificity (93.33%), and overall accuracy (84.7%)

According to ROC analysis, REMS and SOFA scores were significantly valid in predicting mortality (*p* < 0.001). Although the SOFA has good discriminatory power for mortality likelihood (AUC = 0.850), the REMS at cutoff > 3 exhibited excellent discriminatory power (AUC = 0.918) and conveyed a higher specificity (97.2%) with 91.8% overall accuracy. Conversely, the SOFA score had excellent power for predicting complications (AUC = 0.913), with 78.57% sensitivity, 93.33% specificity, and 84.7% overall accuracy at a cutoff > 1 (Table 6 and Figs. 4 and 5).

**Table 6:** Best cutoff values, sensitivity, specificity, and AUC of significant predictors for mortality and complications in acute paraphenylenediamine-poisoned patients

Adverse outcomes	AUC	95% CI	*p-*value	Cutoff value	Sensitivity (%)	Specificity (%)	PPV (%)	NPV (%)	Accuracy (%)
Mortality									
REMS	0.918	0.844–0.964	< 0.001*	> 3	76.0	97.2	90.5	92.1	91.8
SOFA score	0.850	0.763–0.914	< 0.001*	> 3	84.0	75.0	53.8	93.1	77.3
Complications									
SOFA score	0.913	0.823–0.967	< 0.001*	> 1	78.57	93.33	94.3	75.7	84.7

*AUC* area under the curve, *CI* confidence interval, *PPP* positive predictive value, *NPP* negative predictive value, *REMS* Rapid Emergency Medicine Score, *SOFA* Sequential Organ Failure Assessment, *significant at *p* < 0.05

## Discussion

Acute PPD poisoning is an emerging health problem causing deleterious adverse outcomes worldwide, especially in developing countries. The current study revealed that the highest percentage of acute PPD were females (60.8%) with a median age of 23 (IQR: 19–31), and most cases (95.9%) occurred as suicidal attempts. Similarly, Filali et al. (2006), Elgamel and Ahmed (2013), Khan et al. (2016), and Khaskheli et al. (2018) reported female predominance in the younger age group for suicidal intention. Younger females are more susceptible to acute PPD poisoning because they commonly use PPD-based henna and hair dyes for cosmetic purposes such as enhancing hair color and tattooing. Additionally, females are emotionally sensitive and more vulnerable to suicide (Ishtiaq et al. 2017). Because of the widespread availability of PPD and affordable pricing make it the best way to commit suicide without arousing suspicion (Tsirigotis et al. 2011).

Although most non-survivors were significantly males (60%) with a higher median age than survivors (28 vs. 22 years), the age and sex distribution were not significantly associated with complicated outcomes. Partially agreeing with the current study, Sanchez et al. (2016) reported a higher mean age of non-survivors (34.3 ± 12.9 years) than survivors (22.7 ± 6.7) with no significant association with gender. However, Haider et al. (2018) found that the male gender was an independent mortality predictor of PPD poisoning (*p* = 0.001). The predominance of males among non-survivors can be explained by the “gender paradox” in suicide, where males are more likely to commit suicide two to three times than females. In contrast, females primarily attempt suicide and survive (Bhagavathula et al. 2019).

Our study demonstrated a significant association between the prolonged delayed period > 6 h before hospital admission and all adverse sequences (*p* < 0.001). Likewise, Zaghla et al. (2019) reported that a prolonged prehospitalization period (> 6 h) was significantly associated with mortality (*p* = 0.003) and the development of acute lung injuries (*p* = 0.001) in acute PPD-poisoned patients. Accordingly, Balasubramanian et al. (2014) demonstrated that prolonged delay till hospitalization increases clinical severity and postpones the emergent therapy, aggravating the mortality risk in acute PPD-intoxicated cases.

Acute PPD poisoning exhibits a biphasic course. The first phase starts within 4–6 h post-ingestion and is characterized by throat and mouth burning sensation, numbness, intense facial and upper airway edema, and persistent vomiting with dehydration (Chaudhary et al. 2013). Fortunately, if the patients survive, they pass to the second phase, which may occur after 12 h and last from days to weeks (Anuradha et al. 2004; Chaudhary et al. 2013). Rhabdomyolysis, acute tubular necrosis, and renal failure are the leading delayed complications in the later phase (Anuradha et al. 2004; Ali et al. 2020).

Accordingly, many previous studies emphasize that a triad of cervicofacial edema with stridor, rhabdomyolysis with cola-colored urine, and acute renal failure confirms acute PPD poisoning even without supporting exposure history and laboratory investigations (Ishtiaq et al. 2017)**.** Similarly, this study demonstrated that GIT manifestations (80.4%), cervicofacial edema (50.5%), dark urine (49.5%), and oliguria (42.3%) were commonly presenting symptoms. The predominance of the GIT manifestations and angioneurotic edema was attributed to the PPD’s chemical properties as a caustic allergic agent (Bhagavathula et al. 2019).

Table 7 shows variant percentages of presenting symptoms, complications, and mortality between our study and previous studies. The contradictory results might be attributed to differences in PPD concentration and ingested amount, sample size, grading severity, delay period before hospital presentation, and treatment modalities among hospitals.

**Table 7:** Comparison of the main presenting symptoms, complications, and mortality between the current study and previous studies of acute paraphenylenediamine poisoning

Study, year	Study location	Sample size	Study period	Age (years)	Main Symptoms	Complications	Mortality
Current study	Tanta, Assiut, and Aswan, Egypt	97	January 2020–January 2022	-Median: 23 (IQR: 19–31) -Range: 18–75	-GIT: 80.4% -CFE: 50.5% -Dark urine: 49.5%	Total: 43.29% -ARF: 38.14% -ELE: 37.11% -Rhabdomyolysis: 21.64%	Total: 25.77% *Causes:* -RF: 10.30% -Multifactorial cause: 8.24% -Cardiac cause: 4.12% -ARF: 3.09%
Gayathri et al. 2021	Secunderabad, India	42	July 2018–2020	-Mean ± SD: 24 ± 6 -Range: 18–45	-Pain in the oral cavity: 95% -Vomiting: 90% -CFE: 85.7% -Stridor: 83% -Dark urine: 80% - Myalgia: 76%	-Rhabdomyolysis: 80.9% -ARF: 19%	Total: 4.7% *Causes:* -Respiratory complications -ARF
Kumar et al. 2020	Andhra Pradesh, India	106	June 2019–November 2019	-Range: 10–60	-Dysphagia: 67.9% -CFE: 66% -Stridor: 47.1% -Dark urine: 47.1% -Myalgia: 35.8%	-Respiratory distress: 16.9% -Acute renal injury: 24.5%	Total: 11.3%
Ali et al. 2020	Aswan, Egypt	94	January 2019–December 2019	-Mean ± SD: 26.1 ± 12.9	-CFE: 85.1% -Difficulty in opening the mouth: 73.4% -Dark urine: 50% -Muscle weakness: 35.2% -Laryngeal edema: 28.7%	-Acute kidney injury: 29.7% -Acute liver injury: 2.1%	Total: 27.7% *Causes*: -RF: 8.5% -Myocarditis or fatal arrhythmia: 7.5% -Sepsis: 7.5% -ARF: 4.3%
Zaghla et al. 2019	Luxor, Egypt	40	January 2016–July 2017	-Mean ± SD: 25 ± 11 -Range: 5–57	-Black urine: 100% -Cyanosis: 85% -Myalgia: 77.5% -Angioedema: 67.5%	-Rhabdomyolysis: 77.5% -Acute lung injuries: 50%	Total: 37.5% *Causes*: -Multiorgan failure
Haider et al. 2018	Multan, Pakistan	32	September 2016–April 2017	-Mean ± SD: 21.06 ± 3.25 -Range: 16–25	-Difficulty in mouth opening: 93.8% -CFE: 93.8% -Oral erythema: 81.3% -Sore throat: 75%	-Rhabdomyolysis: 46.9% -ARF: 34.4% Hemodynamic shock: 28.1% -Acute hepatitis: 18.75%	Total: 28.1%
Khaskheli et al. 2018	Nawabshah, Pakistan	1032	January 2011–December 2016	-Mean ± SD: 22.08 ± 8.42 -Range: 12–40	-Dysphagia: 100% -CFE: 90.99% -Dyspnea: 89.82% -Dark urine: 75.19% -Myalgia: 68.99%	-ARF: 58.46%	Total: 31.67% *Causes:* -Cardiotoxicity -ARF
Tanweer et al. 2018	Multan, Pakistan	122	April 2015–September 2016	-Mean ± SD: 23.21 ± 8.2	-CFE: 95% -Dark urine: 77.9%	-Rhabdomyolysis: 74.5% -Acute kidney injury: 30.3% -Myocarditis: 27%	Total: 28% *Causes:* -Ventricular arrhythmias: 73.5% -ARF: 14.7% -Asphyxia: 5.88% -Aspiration pneumonia: 5.88%
Manzoor et al. 2017	Rahim Yar khan, Pakistan	60	July 2016–December 2016	-Mean ± SD: 25.42 ± 7.42 -Range: 12–60	-Dysphagia: 73.33 -CFE: 40% -Hyperkalemia: 53.33%	-ARF: 26.67% -Acute hepatitis: 13.33%	-------
Ishtiaq et al. 2017	Southern Punjab, Pakistan	109	2015	-Mean ± SD: 22 ± 3.4	-Dark urine: 100% -Oligouria: 95% -CFE: 95% -Dysphonia: 95% -Throat pain: 88%	-Rhabdomyolysis: 86% -ARF: 63% -Acute hepatitis: 51%	Total: 36% *Causes:* -Acute hepatitis - ARF
Ramulu et al. 2016	Hyderabad, Telangana, India	31	November 2011–October 2013	-Mean ± SD: 24 ± 8 -Range: 16–45	-Myalgia: 90.3% -CFE: 83.8% -Vomiting: 80.6% -Dark urine: 58%	-Rhabdomyolysis: 80.9% -ARF: 19%	Total: 12.9% *Causes:* -RF - Pneumonia - ARDS -Sepsis
Khan et al. 2016	Dera Ismail Khan, Pakistan	38	2013–2015	-Mean ± SD: 22.08 ± 6.4 -Range: 15–45	-Dysphagia: 100% -CFE: 94.7% -Dyspnea: 94.7% -Dark urine: 21.1%	-Rhabdomyolysis: 57.9% -ARF: 39.5%	Total: 47.4 *Causes* -RF
Tiwari et al. 2016	Gwalior, India	50	August 2008-October 2009	- More patients in the age group 15–25	-Angioneurotic edema: 42% -Oliguria: 38%	-Acute kidney injury: 38% -Acute liver injury: 18%	Total: 20% *Causes:* -Cardiotoxicity
Mohammed et al. 2015	Khartoum, Sudan	40	May 2013–December 2014	More patients in the age group more than 32	-Neck swelling: 22.5% -Muscular Edema: 15%	-Rhabdomyolysis: 75%	-------
Mahsud 2015	Dera Ismail Khan, Pakistan	38	September 2013–September 2015	-Mean ± SD: 22.08 ± 6.42 -Range: 15–45	- Laryngeal edema: 92.1%	-------	Total: 39.45% *Causes:* -Asphyxia and RF
Sakuntala et al. 2014	Hyderabad, India	31	November 2011–October 2013	-More patients in the age group 21–30	-Vomiting and dysphagia: 80% -Dark urine: 58% -Angioedema: 48.38%	-Rhabdomyolysis: 80.9% -ARF: 19.3%	Total: 12.9% *Causes:* -Pneumonia -Sepsis -ARF
Shigidi et al. 2014	Khartoum, Sudan	30	April 2012–March 2013	-Mean ± SD: 25.6 ± 4.2	-Oligaura: 90% -Dark urine: 76.7% -Angioneurotic edema: 53.3%	-Acute kidney injury: 86.7%	Total: 3.3%
Elgamel and Ahmed 2013	Khartoum, Sudan	200	June 2008–December 2008	-More patients in the age group 10–20	-Vomiting: 31% -Angioneurotic edema: 22.5% -Dyspnea: 18%	-Respiratory problems: 22.5% -Acute kidney injury: 20.5% -Cardiac problems: 1%	Total: 3.5% *Causes:* -Respiratory obstruction -ARF
Jain et al. 2011	Jhansi, India	1020	July 2004–March 2009	-More patients in the age group 15–25	-CFE: 73.03% -Dysphagia: 71.17% -Stridor: 22.45% -Dark urine: 53.82% - Myalgia: 47.05%	-Rhabdomyolysis: 53.82% -Acute renal failure: 25.88%	Total: 22.48% *Causes:* -Acute cardiac injury and myocarditis
Shalaby et al. 2010	Ain Shams, Egypt	25	2001–2008	-Mean ± SD: 35.34 ± 10.5	-CFE and laryngeal edema (72%)	-Rhabdomyolysis: 100% -Acute kidney injury: 80% -Acute liver injury: 76%	Total: 16% *Causes:* -Ventricular arrhythmia

*SD* standard deviation, *IQR* interquartile range, *GIT* gastrointestinal, *CFE* cervicofacial edema, *ARF* acute renal failure, *ELE* elevated liver enzymes, *RF* respiratory failure, *ARDS* acute respiratory distress syndrome

The current study revealed that non-survivors had significantly cervicofacial edema and respiratory distress manifestations as well as low SBP, DBP, and abnormal ECG than survivors. Consequently, the prominent causes of death were respiratory failure, multifactorial, and cardiac injury (10.30%, 8.24%, and 4.12%, respectively), which are consistent with Mahsud (2015), Khan et al. (2016), Zaghla et al. (2019), and Ali et al. (2020). Severe cervicofacial edema compromises the airway causing severe hypoxemia and respiratory failure (Ishtiaq et al. 2017; Lohano et al. 2017). Additionally, Ashraf et al. (1994) revealed that initial hypotensive shock was a poor outcome predictor in patients with acute PPD poisoning.

Although the clear mechanism of PPD-induced cervicofacial edema and respiratory symptoms is not fully understood, it could be explained by the formation of quinondimine oxidative PPD metabolite, which is responsible for intense mucous membrane irritation (Hamdouk et al. 2008). According to some authors, PPD angioneurotic edema is caused by blood colloid affection or changes in vascular permeability through parasympathetic nervous system involvement (Zaghla et al. 2019). Another justification is that the oxidized trimer metabolite (BB) may potentiate systemic anaphylaxis (Elgassim et al. 2022).

Besides, many previous studies described cardiotoxicity in acute PPD poisoning, including myocarditis, myocardial infarction, ventricular thrombus formation, and cardiac dysrhythmias (Zeggwagh et al. 2003; Brahmi et al. 2006). Ventricular and supraventricular ectopics, bundle branch block, and ST-T changes are commonly reported ECG findings (Chaudhary et al. 2013). The post-mortem cardiac examination confirmed that PPD-induced myocardial rhabdomyolysis is a reliable mechanism for causing cardiogenic shock (Ababou et al. 2000; Shalaby et al. 2010). Accordingly, Jain et al. (2011) and Tanweer et al. (2018) reported that myocarditis and ventricular arrhythmias were the leading causes of death (28.15% and 73.5%, respectively) in acute PPD poisoning.

Our study demonstrated that the complicated group had significantly more GIT symptoms, dark urine, oliguria, higher SBP, and metabolic acidosis, as well as elevated serum CPK, AST, ALT, blood urea, and serum creatinine levels than the improved group. Correspondingly, forty-two patients developed delayed sequels, including acute renal failure, elevated liver enzymes, and rhabdomyolysis (38.14%, 37.11%, and 21.64%, respectively). Similarly, Khan et al. (2016), Tiwari et al. (2016), and Tanweer et al. (2018) reported comparable renal failure incidences (39.5%, 38%, and 30%, respectively). However, Shalaby et al. (2010), Shigidi et al. (2014), and Khaskheli et al. (2018) reported higher renal failure frequencies (80%, 86.7%, and 58.46%, respectively).

Various mechanisms have explained acute renal failure following acute PPD poisoning. The PPD and its oxidant metabolites (quinone-diamine) have direct nephrotoxic effects on renal parenchyma (Akl and Alturki 2018). Synergistically, PPD-inducing rhabdomyolysis, hemolysis, and methemoglobinemia contribute to renal tubular damage (Shigidi et al. 2014).

The PPD induces rhabdomyolysis by stimulating calcium release from the smooth endoplasmic reticulum causing continuous muscular contractions and irreversible necrosis (Chrispal et al. 2010). The experimental study by de Mas et al. (2017) unveiled the metabolic effects of PPD-induced rhabdomyolysis by inhibiting glycolysis and glycogen turnover, resulting in decreased adenosine triphosphate (ATP) synthesis and cellular apoptosis. Additionally, diffuse muscle tissue swelling causing pressure on blood vessels and resulting in muscle injury is another postulated mechanism of rhabdomyolysis (Shalaby et al. 2010).

Although the mechanism of PPD-induced hepatotoxicity is still not fully concluded, PPD exerts its toxic effects directly or after oxidation in the liver. Additionally, extrahepatic PPD activation to free aminyl radial covalently binds with thiol, resulting in a more potent hepatotoxic compound (Shalaby et al. 2010). Although an experimental study by Ghonemy et al. (2021) recorded hepatic histopathological improvement after two weeks of PPD cessation, Ishtiaq et al. (2017) reported that PPD-induced acute hepatitis was the leading cause of mortality (20 of 56 patients; 35.7%).

The current study recorded 34.0% underwent tracheostomy, 38.14% received hemodialysis, and 64.9% were admitted to the ICU. Likewise, Gayathri et al. (2021) reported that 38% of acute PPD patients required tracheostomy. Inconsistent findings were observed, where Tanweer et al. (2018) reported higher rates of tracheostomy (82%) and hemodialysis (43.2%), and Elgamel and Ahmed (2013) revealed lower tracheostomy and dialysis incidences (18% and 20%, respectively).

As a result of the preponderance of acute respiratory and renal failure for unfavorable outcomes, our study found a significant association between mortality and tracheostomy as well as complications with hemodialysis. In addition, the considerably high ICU admission incidence was attributed to respiratory failure, unresponsive hypotension, and cardiac arrhythmias that require close hemodynamic monitoring and intensive management (Elgazzar et al. 2021; El-Sarnagawy et al. 2022b).

Scoring systems are simple, reliable tools for predicting patient outcomes based on available clinical and laboratory data. This study represents the initial evaluation of the accuracy of emergency and physiological scores in anticipating various PPD poisoning outcomes.

Although the current study revealed that the medians of REMS and SOFA scores were significantly higher in non-survivors than survivors (*p* < 0.001 for each), REMS had the highest odds (OR = 1.91 [95% CI: 1.41–2.60], *p* < 0.001) by multivariate analysis. Additionally, ROC curve analysis demonstrated that REMS at cutoff > 3 exhibited an excellent discriminatory power (AUC = 0.918), high accuracy (91.8%), and conveyed a better specificity (97.2%) for mortality compared with the SOFA score.

Likewise, Abd Elghany et al. (2018) demonstrated that REMS at cutoff level ≥ 4.5 had an excellent discriminatory power (AUC = 0.970) for predicting aluminum phosphide mortality with 89.3% sensitivity and 95.5% specificity with no significant difference with AUC values of APACHE and SOFA scores. Shama et al. (2021) revealed that REMS had significantly better performances (AUC = 0.868) than Acute Physiology and Chronic Health Evaluation (APACHE) II score and SOFA scores in predicting organophosphate (OP) poisoning mortality at cutoff level > 9.0 with 75.0% sensitivity, 94.57% specificity, and overall accuracy of 85%.

Moreover, the current study highlights the significant role of SOFA score in predicting PPD adverse sequels by having the highest odds ratio compared to other significant variables (OR = 4.97 [95% CI: 1.16–21.21], *p* = 0.001). Additionally, it had good to excellent power of discrimination for predicting mortality (AUC = 0.850) and adverse sequel (AUC = 0.913) at cutoff values > 3 and > 1, respectively. Similarly, Zaghla et al. (2019) verified that SOFA score > 3 was significantly correlated with survival (*p* < 0.001) and can predict morbidity and acute lung injury in patients with acute PPD poisoning.

Furthermore, Ebrahimi et al. (2018) revealed that the SOFA score was the most predictive for short-term clinical outcomes of acutely poisoned patients, with the highest accuracy (86.2%) and AUC = 0.897 than PSS and APACHE IV. Moussa et al. (2018) observed that SOFA score > 5 had the highest accuracy (99.5%) and predictive ability (AUC = 0.995) compared with APACHE II score and serum amylase in predicting the OP poisoning poor outcomes. Similarly, Sharif and Fayed (2021) and Sharif et al. (2021) reported that SOFA > 4.5 had an excellent discriminatory power for predicting unfavorable outcomes in patients with acute hydrogen cyanamide and methanol poisoning (AUC = 0.961 and 0.955, respectively).

The superiority of REMS over SOFA score in mortality prediction of acute PPD poisoning as its evaluation is based mainly on the cardiorespiratory and neurological status. The absence of hepato-renal components in the REMS score could explain the lack of its significant association with complicated outcomes. Conversely, the SOFA score can significantly predict mortality and complications by simultaneously evaluating respiratory, cardiovascular, renal, hepatic, hematological, and neurological systems. Unfortunately, we observed lower REMS and SOFA values predicting PPD adverse outcomes than in other toxicological studies, which necessitate more intensive management and follow-up.

## Strengths and limitations

Predicting the multiple potential PPD outcomes using scores based on accessible clinical and laboratory investigations strengthens the current study’s practicability. Although the study’s sample size was relatively small with a retrospective design, the multicenter retrieved data increased the reliability and applicability of the study findings. We could not estimate the ingested PPD amount, which significantly influences poisoning severity. Likewise, we could not investigate the impact of molecular markers in predicting the adverse consequences of acute PPD poisoning due to their unavailability in emergency hospitals and the study’s retrospective nature. Moreover, the absence of the follow-up of liver and kidney status after patients’ discharge impeded the ultimate outcome assessment.

## Conclusion

Acute paraphenylenediamine poisoning is a life-threatening emergency causing significant morbidity and mortality. It was commonly observed among young females for suicidal intentions. The most frequent presenting symptoms were gastrointestinal manifestations, cervicofacial edema, dark urine, and oliguria. Respiratory failure was the leading cause of mortality, whereas acute renal failure was the chief complication. The delay time for admission, abnormal ECG, initial serum CPK, and HCO_3_ levels, as well as REMS and SOFA scores, were independent predictors of acute PPD poisoning adverse outcomes. Although the REMS more accurately predicted mortality, the SOFA score was the main determinant of complications development.

## Recommendations

- All acute PPD-poisoned patients should be initially assessed according to REMS and SOFA scores to stratify high-risk patients for adverse outcomes.
- Further prospective studies testing the score’s validity in acute PPD poisoning are highly recommended.
- Educational programs to raise public awareness as well as restrict regulation and sales of PPD are urgently needed to alleviate this problem.

## Data Availability

The datasets used and/or analyzed during this study are available from the corresponding author on reasonable request.
